# TMPL: a database of experimental and theoretical transmembrane protein models positioned in the lipid bilayer

**DOI:** 10.1093/database/bax022

**Published:** 2017-03-24

**Authors:** Guillaume Postic, Yassine Ghouzam, Catherine Etchebest, Jean-Christophe Gelly

**Affiliations:** 1 Inserm U1134, Paris, France; 2 Université Paris Diderot, Sorbonne Paris Cité, UMR_S 1134, Paris, France; 3 Institut National de la Transfusion Sanguine, Paris, France; 4 Laboratory of Excellence GR-Ex, Paris, France

## Abstract

Knowing the position of protein structures within the membrane is crucial for fundamental and applied research in the field of molecular biology. Only few web resources propose coordinate files of oriented transmembrane proteins, and these exclude predicted structures, although they represent the largest part of the available models. In this article, we present TMPL (http://www.dsimb.inserm.fr/TMPL/), a database of transmembrane protein structures (α-helical and β-sheet) positioned in the lipid bilayer. It is the first database to include theoretical models of transmembrane protein structures, making it a large repository with more than 11 000 entries. The TMPL database also contains experimentally solved protein structures, which are available as either atomistic or coarse-grained models. A unique feature of TMPL is the possibility for users to update the database by uploading, through an intuitive web interface, the membrane assignments they can obtain with our recent OREMPRO web server.

## Introduction

Transmembrane proteins are quantitatively important biological molecules which play various and central roles in numerous physiological processes, essentially as transporters, receptors and enzymes. The study of their function is critical for many industrial and medical applications, such as the development of novel drugs. However, understanding the function of these fat-soluble proteins is hampered by the technical difficulty of determining their three-dimensional structure and, to a greater extent, by the high difficulty of experimentally observing the orientation of the protein structure in the lipid bilayer. Indeed, proteins for which both the tilt angle and the hydrophobic thickness have been determined can literally be counted on the fingers of one hand (1). Finding the theoretical inclination of a protein structure in the membrane is not trivial (the molecular axis may greatly differ from the normal of the bilayer planes), but it can still be achieved by using the different positioning algorithms that have been developed for this purpose (1–5).

**Figure 1.**
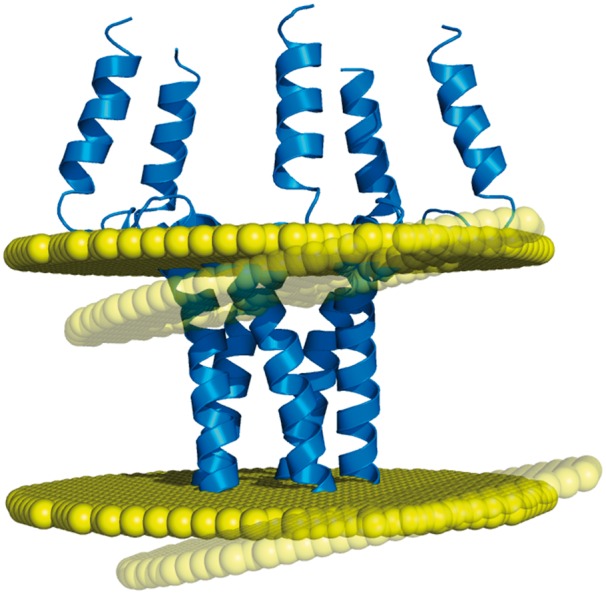
Example of discrepancy between different membrane positioning methods. The homopentameric structure of the phospholamban (PDB code: 1zll) is differently oriented in the membrane by OPM and Ez-3 D algorithms (opaque and transparent bilayer planes, respectively), with a helical tilt difference of 8.5° (1).

Based on such algorithms, the PDBTM (6) and OPM (5) databases have remained for a decade the only two resources of membrane assignments. This low number of databases is problematic regarding the fact that transmembrane protein structures may actually have different but equally valid orientations in the lipid bilayer, due to the orientational disorder that exists in the biological membranes (7). For example, the transmembrane domain of the phospholamban (PDB code: 1zll) has a measured helical tilt which varies from 10° to 28° depending on the studies, the latest of which reports a value of 13°, with an experimental error of 4° (8). Thus, it is certainly no coincidence that different algorithms produce different membrane orientations for this protein structure (Figure 1). For such cases, an additional point of view appears necessary for providing an alternative to the membrane assignments from OPM and PDBTM. In addition, another important need has to be met regarding the coarse-grained models of proteins, since they are included in none of the two existing databases, despite the popularity of this structural representation. While the recent MemProtMD (9) does include coarse-grained models of transmembrane proteins, this database belongs to a different category from OPM and PDBTM, since its entries are protein structures inserted into an explicit membrane environment. Although this representation can reveal local bilayer deformations, MemProtMD cannot provide any delimitation of the transmembrane segments (nor any calculations of the tilt angles and hydrophobic thickness), as do OPM and PDBTM with their planar bilayers.

One may regret that none of the existing databases includes predicted structures of transmembrane proteins, especially when considering (i) the few native structures available for these proteins, (ii) the steady progress in protein structure modeling and (iii) the growing number of theoretical models available in specialized databases, such as ModBase (10) and SWISS-MODEL (11). Also regrettable is the lack of quality assessment for the orientation of the structures in the lipid bilayer. Indeed, the membrane assignments found in the existing databases are actually predictions made by computational methods—sometimes confirmed by the very few experimental data available—the reliability of which needs to be evaluated. This can be achieved with different types of scoring functions, in particular those that have been recently developed for membrane protein structures (12–14).

In this report, we present TMPL, a new database of transmembrane protein models positioned in the lipid bilayer. The database includes atomistic and coarse-grained protein models—such as those used with the MARTINI force field (15)—oriented in the membrane by the ANVIL algorithm (2), which has been designed to process both representations of protein structure. The protein structures in TMPL are either experimental or theoretical models, whose quality is assessed with the membrane-specific statistical potential MAIDEN (12). This inclusion of predicted structures makes TMPL a large database, with >11 000 entries at its launch. The TMPL database is expected to grow along with the number of native and predicted structures available, and through its interfacing with the OREMPRO web server (16) (a pipeline of the ANVIL algorithm and the MAIDEN method), which allows users to deposit their own models.

## Construction and content

### Sources of protein structures

**Figure 2.**
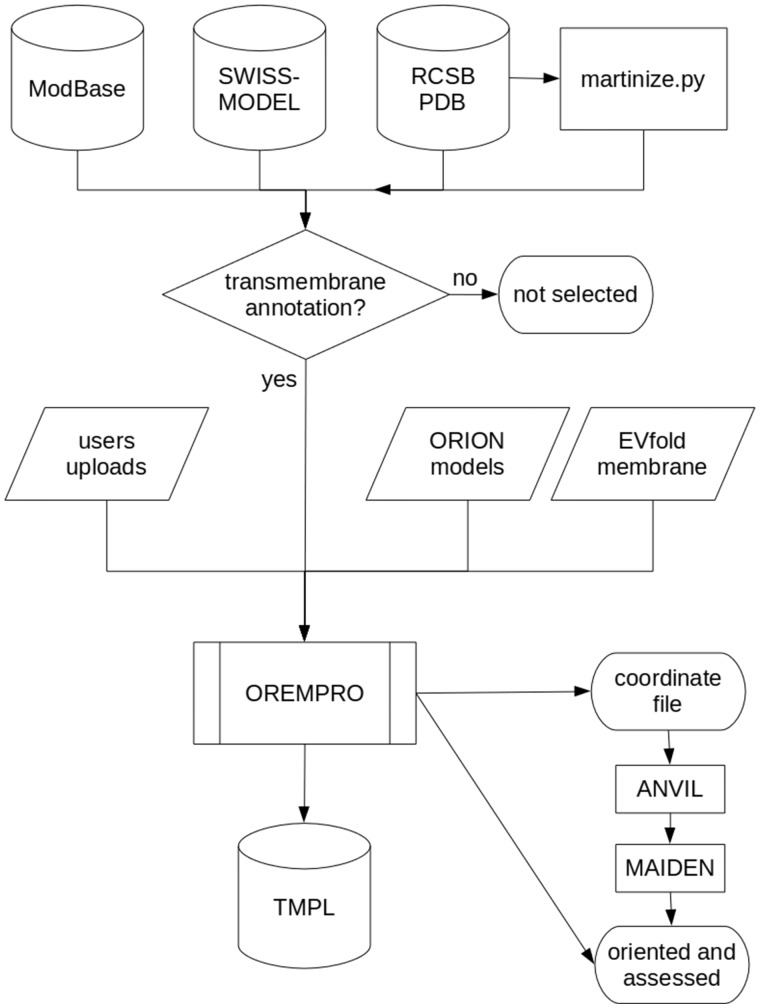
Flowchart describing how the TMPL database is generated. Predicted, native, and ′martinized′ structures from other databases are selected for being annotated as ′transmembrane′ proteins, before being submitted to the OREMPRO server. The latter is a pipeline of the ANVIL and MAIDEN methods, which are aimed at the orientation and assessment of transmembrane protein structures, respectively.

Most of the protein structures in TMPL come from external databases and were selected for containing the term ′transmembrane′ in either their keywords, title or description (Figure 2). Experimental models were downloaded from the RCSB PDB (www.rcsb.org), while theoretical models generated by comparative modeling were downloaded from ModBase (modbase.compbio.ucsf.edu) and SWISS-MODEL (swissmodel.expasy.org) databases. Additionally, *de novo* predicted structures were taken from the top-ranked models of the EVfold_membrane dataset (17). For all the theoretical models, secondary structures were assigned with the DSSP algorithm (18). The coarse-grained models in TMPL were generated from experimental structures of transmembrane proteins, by using the ′martinize.py′ script (19). Thus, the coordinate files of coarse-grained structures are compatible with the MARTINI force field and describe the spatial arrangement of backbone beads (BB) and side-chain beads (SC1 to SC4).

Besides the aforementioned databases, TMPL has two minor—yet qualitatively important—sources of transmembrane protein models. The first of these sources is constituted by the structures that users can deposit through the TMPL website. Indeed, an original feature of TMPL is the possibility for users to upload protein models and their membrane assignments to the database. This is made possible by the connection between the TMPL database and our OREMPRO web server for orienting protein structures into the lipid bilayer. As an example, the first model deposited in this way is a theoretical model of the human red blood cell DARC protein, generated in our laboratory (20), and whose native structure still remains unsolved. The other source of models are the predicted structures that we generate using ORION (21), our web server dedicated to template-based modeling, which has recently been improved (22). The pipeline has not yet been developed for a high-throughput production of protein models, but ORION has been shown to produce structures of good quality at a fairly high rate, during the eleventh Critical Assessment of Structure Prediction experiment (under the name ′Alpha-Gelly-Server′).

### Membrane assignment and quality assessment

The transmembrane segments of all the models in TMPL have been delimited by using our algorithm for positioning protein structures in the lipid bilayer, named ANVIL, which is capable of processing both atomistic and coarse-grained representations of protein structure. This computational method is based on a membrane propensity scale, which by default is binary (a value of +1.0 or −1.0 is attributed to each residue type, depending on its tendency to be located inside or outside the membrane, respectively) but can be modified on the OREMPRO web server. For all the membrane assignments in TMPL that have been automatically generated, the default membrane propensity scale has been used. When a user-deposited structure is oriented using a different scale, the modified propensity values is recorded in the TMPL database and displayed on the protein entry page (see online supplementary material for Figure S1). Finally, the membrane assignments produced by ANVIL are based either on the sole membrane propensity scale, or on an additional geometric criterion (16). The latter has been developed to improve the membrane positioning for certain types of structures, particularly the beta-barrel proteins. It consists in making the sphere—centered on the protein center of mass—that is used to define the exploration axes smaller than the protein structure (rather than large enough to contain it). In this way, the resulting membrane planes are constrained to not deviate too much from the protein center of mass. For all oriented structures in TMPL, both approaches have been followed; however, the orientations obtained with the additional geometric criterion have been retained only when they had a higher number of transmembrane segments. It must be noted that monotopic segments (i.e. those which do not completely span across the membrane) are not counted as transmembrane, unlike in other databases (see online supplementary material for Figure S1).

After a protein model is oriented in the lipid bilayer, the structural quality of the transmembrane domain is assessed by using the MAIDEN pseudo-energy function (12). This scoring function is a statistical potential of mean force (23) that has been trained on a dataset of native transmembrane protein structures. The MAIDEN score (or pseudo-energy) is aimed at evaluating the relative structural quality, which means that it can be used to rank several protein models by their quality. For each protein structure in TMPL, the attributed MAIDEN score comes with a color code, which is derived from the Z-score of the pseudo-energy (16). This provides a qualitative evaluation of the absolute quality of the model (from red for bad models, to blue for native or near-native structures). Finally, the use of MAIDEN also offers an assessment for the quality of the orientation calculated by ANVIL; e.g. a model of high quality with a poor membrane assignment will have a relatively high (i.e. bad) MAIDEN score with a red color.

### Entry codes in TMPL

Every protein structure in TMPL has a unique accession code, whose pattern is dependent on whether the entry is a theoretical or an experimental model. The entry codes of native structures—and their ′martinized′ versions—include the PDB ID, which is followed by the representation ('AT' for atomic, or ′CG′ for coarse-grained), and ends by ′D′ (default) or ′G′ (geometric), this terminal letter corresponding to the methods used by the orientation algorithm to determine the membrane boundaries. Thus, the pattern of the accession codes for native structures can be written as: TM[PDB ID][AT|CG][D|G]. For predicted structures, the entry code includes the UniProtKB AC of the protein chain, the source of the model, i.e. ′S′ (SWISS-MODEL), ′M′ (ModBase), ′O′ (ORION), ′E′ (EVfold), or ′U′ (user-deposited), the number of the model (from 1 to 99; because there may be more than one model of the same protein), and the positioning method employed by ANVIL ('D' or ′G′). Thus, the pattern for predicted structures is: TM[UniProtKB AC][S|M|O|E|U][1-99][D|G]. Finally, when several valid orientations are available, a number is appended to their accession codes to distinguish them (see discussion).

### Database updates

Thanks to the automated procedure for generating TMPL, our database will be regularly updated, with a frequency that depends on the source of protein models. Thus, every 6 months the PDB, ModBase and SWISS-MODEL databases will be searched for new structures of transmembrane proteins, which will constitute a major update. Smaller and more frequent updates of our database will be performed for every predicted structure that we will produce with ORION, and for every user-deposited model. Finally, the entries in TMPL may be subject to change, if any correction appears necessary. In such a case, the old entry page and its content will be superseded and unreferenced, while being still accessible through the new entry page. Of course, the creation date of each entry in TMPL is recorded and displayed on its page.

## Utility and discussion

### Search and browse TMPL

The protein structures in TMPL can be accessed by filling a search form with any of several data types: the protein name or any keywords from its description, the organism (genus and/or species), the type of model (experimental or theoretical), the structural representation (atomistic or coarse-grained), or the type of transmembrane protein structure (α-helical or β-sheet). Users can also search TMPL by using entry codes from UniProt, PDB and Pfam databases. A quick search can be performed from anywhere in the website, by using the text area of the TMPL navigation bar, and will match protein keywords, organism, or entry codes. Alternatively to the search, our database can be browsed by either protein families or organisms. The protein families correspond to the Pfam families to which belong the transmembrane domains of the protein structures in TMPL. The other browsing option consists in an interactive phylogenetic tree displaying all the taxonomic ranks, with each terminal taxon being a hyperlink to the corresponding TMPL entries.

### Information and interactive visualization

**Figure 3.**
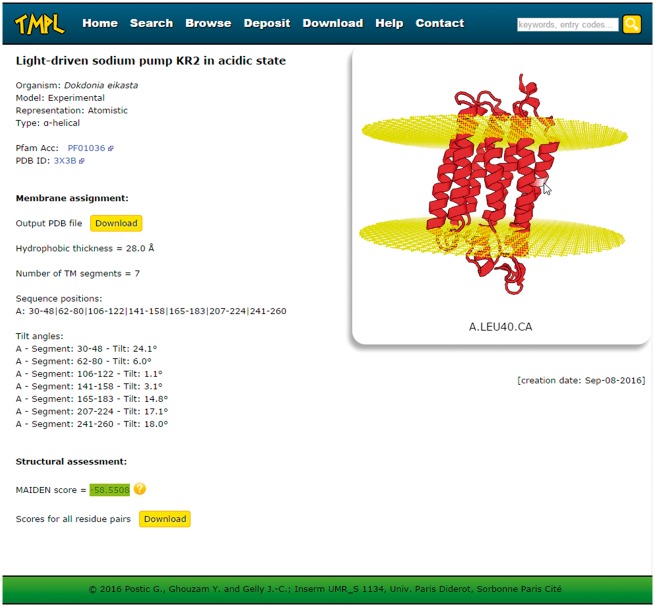
Screenshot of a TMPL entry page. Left: information about the protein structure, the membrane assignment, and the structural assessment. Right: interactive visualization of the protein structure with the membrane planes (represented by two grids of atoms). Top: the navigation bar, with the different sections of the website, and the ′quick search′ text area.

Every entry page displays the following information about the protein structure (Figure 3): the name or title of the structure, the organism, the representation, the type of transmembrane protein, the Pfam Acc (if any), the PDB ID or UniProtKB AC (depending on whether the model is experimental or theoretical, respectively), the membrane assignment by ANVIL, the structural assessment by MAIDEN, and the creation date. The membrane assignment is itself composed of the number, sequence positions and tilt angles of the transmembrane segments and the hydrophobic thickness. The protein structure and the membrane planes can be visualized directly on the web page, thanks to the WebGL protein viewer PV (24), which offers an interactive visualization of protein 3 D models represented as ribbon diagrams (or spheres for the coarse-grained models). The coordinate file of the structure and its assigned membrane can be downloaded to be visualized with any locally installed molecular viewer. A second download link gives access to the details (as a text file) of the structural assessment performed with MAIDEN. Finally, for all entries, these PDB and text format files are gathered in a compressed archive available on the ′Download′ page of TMPL.

### Users depositions and alternative assignments

Allowing users to deposit models in TMPL—which is made easy by the connection with our OREMPRO web server—has a 2-fold purpose: (i) completing the database with native or predicted protein structures that have not yet been included and (ii) uploading structures of transmembrane proteins that are already part of TMPL, but with an alternative membrane assignment. Indeed, the inclination of a protein in a biological membrane is not a constant value, due to the thermodynamic entropy of the lipid bilayer environment (7), hence the possibility for a given structure to have different, yet equally valid, membrane assignments. For example, the native structure of the bacteriorhodopsin monomer (PDB 1py6) has two plausible orientations in the lipid bilayer, as we have previously shown (2), and both are included in TMPL as distinct entries. In this case, the positioning depends on the input coordinate file: entry ′TM1PY6ATD1′ is the membrane assignment of the bacteriorhodopsin taken from the RCSB PDB, whereas ′TM1PY6ATD2′ corresponds to the structure of the same protein, but taken from the OPM database and oriented in the membrane using OREMPRO. Some protein structures from OPM differ from their PDB counterparts in that they have been subjected to structural adjustments (5) (never reported on OPM entry pages), which may impact the assignments produced by OREMPRO. In the case of 1py6, these modifications bring about an improvement: the transmembrane domain has 7 membrane-spanning helices (instead of 5) and a better MAIDEN score. For further updates of TMPL, we will consider a systematical use of these structural adjustments, which can be performed by various external methods, such as the web servers PPM (5), PDB_Hydro (25), and PDB_REDO (26). Other cases of alternative assignments may result from the use of different membrane propensity scales for a given protein structure. Although we used the same default scale for every model in TMPL, it is unlikely that this single (and binary) scale is most relevant for all proteins, given the diversity of lipid compositions that exists among biological membranes, from one species (or organelle) to another (27). Thus, the fact that TMPL users can deposit relevant orientations that have been produced by tunning the membrane propensity parameters in OREMPRO will help us develop ′organism-specific′ hydrophobicity scales.

### Limitations and perspectives

The protein structures included in TMPL have been selected for having the term ′transmembrane′ in their name or description. The use of this keyword approach is made necessary by the design of our ANVIL algorithm. Indeed, unlike the other automated methods that may fail at orienting predicted transmembrane structures of low quality (thus, identifying them as non-transmembrane proteins), ANVIL has been aimed at systematically positioning the lipid bilayer planes, without trying to discriminate actual transmembrane proteins from others. This means that the current version of ANVIL cannot detect transmembrane proteins and that we have to rely on external annotations for selecting the structures to include in TMPL. As a consequence, TMPL may contain models that do not correspond to actual transmembrane proteins. These are essentially either structural predictions mistakenly annotated as ′putative transmembrane′, or truncated theoretical models of actual transmembrane proteins, e.g. those for which only the non-membrane (globular) domain has been modeled. These ′false positives′ (which can also be found in other databases, e.g. OPM entries 1ipe and 3nw8) will be discarded intermittently—whenever encountered or reported—or systematically, as soon as a new version of ANVIL can detect transmembrane proteins with a high statistical sensitivity, to avoid discarding true transmembrane proteins.

## Conclusions

By including theoretical models for transmembrane proteins of unsolved structures, in addition to atomistic and coarse-grained representations of experimentally solved structures, TMPL fills some of the gaps left by the insufficiently numerous databases of its kind. As a unique feature, TMPL enables users to enrich the database with their own membrane assignments of native or predicted structures, through its easy-to-use web interface and its connection with our OREMPRO web server. Thus, TMPL will centralize our current and future tools for studying transmembrane proteins, as well as our research on high-throughput structural modeling with ORION. This work places itself within the global efforts in structural genomics, focusing on the medically and industrially important transmembrane proteins.

## Availability and requirements

The TMPL database is freely available at http://www.dsimb.inserm.fr/TMPL/. Its web interface is compatible with different browsers (Firefox, Chrome, Opera and Safari), on different operating systems (Windows, Linux and Mac OS) and platforms (though esthetically optimized for laptop screens). The visualization of protein 3 D structures with the PV molecular viewer requires the browser to have WebGL enabled.

## Supplementary data

Supplementary data are available at *Database* Online.

## Supplementary Material

Supplementary Data

## References

[1] Nugent T., Jones D.T. (2013) Membrane protein orientation and refinement using a knowledge-based statistical potential. BMC Bioinform., 14, 276. 10.1186/1471-2105-14-276 PMC3852961 24047460

[2] Postic G., Ghouzam Y., Guiraud V. (2016) Membrane positioning for high- and low-resolution protein structures through a binary classification approach. Protein Eng. Des. Select., 29, 87–92. 10.1093/protein/gzv063 26685702

[3] Tusnády G.E., Dosztányi Z., Simon I. (2005) TMDET: web server for detecting transmembrane regions of proteins by using their 3D coordinates. Bioinformatics, 21, 1276–1277. 15539454 10.1093/bioinformatics/bti121

[4] Schramm C.A., Hannigan B.T., Donald J.E. (2012) Knowledge-based potential for positioning membrane-associated structures and assessing residue-specific energetic contributions. Structure, 20, 924–935. 22579257 10.1016/j.str.2012.03.016 PMC3366090

[5] Lomize M.A., Pogozheva I.D., Joo H. (2012) OPM database and PPM web server: resources for positioning of proteins in membranes. Nucl. Acids Res., 40, D370–D376. 21890895 10.1093/nar/gkr703 PMC3245162

[6] Kozma D., Simon I., Tusnády G.E. (2013) PDBTM: Protein Data Bank of transmembrane proteins after 8 years. Nucl. Acids Res., 41, D524–D529. 23203988 10.1093/nar/gks1169 PMC3531219

[7] Pabst G., Kučerka N., Nieh M.-P. (2014) Liposomes, Lipid Bilayers and Model Membranes: From Basic Research to Application. CRC Press, Boca Raton, FL.

[8] Ghimire H., Abu-Baker S., Sahu I.D. (2012) Probing the helical tilt and dynamic properties of membrane-bound phospholamban in magnetically aligned bicelles using electron paramagnetic resonance spectroscopy. Biochim. Biophys. Acta, 1818, 645–650. 22172806 10.1016/j.bbamem.2011.11.030 PMC3273594

[9] Stansfeld P.J., Goose J.E., Caffrey M. (2015) MemProtMD: automated insertion of membrane protein structures into explicit lipid membranes. Structure, 23, 1350–1361. 26073602 10.1016/j.str.2015.05.006 PMC4509712

[10] Pieper U., Webb B.M., Dong G.Q. (2014) ModBase, a database of annotated comparative protein structure models and associated resources. Nucl. Acids Res., 42, D336–D346. 24271400 10.1093/nar/gkt1144 PMC3965011

[11] Biasini M., Bienert S., Waterhouse A. (2014) SWISS-MODEL: modelling protein tertiary and quaternary structure using evolutionary information. Nucl. Acids Res., 42, W252–W258. 24782522 10.1093/nar/gku340 PMC4086089

[12] Postic G., Ghouzam Y., Gelly J.-C. (2015) An empirical energy function for structural assessment of protein transmembrane domains. Biochimie, 115, 155–161. 26044650 10.1016/j.biochi.2015.05.018

[13] Esque J., Urbain A., Etchebest C. (2015) Sequence–structure relationship study in all-α transmembrane proteins using an unsupervised learning approach. Amino Acids, 47, 2303–2322. 26043903 10.1007/s00726-015-2010-5

[14] Wallner B. (2014) ProQM-resample: improved model quality assessment for membrane proteins by limited conformational sampling. Bioinformatics, 30, 2221–2223. 24713439 10.1093/bioinformatics/btu187 PMC4103597

[15] Marrink S.J., Risselada H.J., Yefimov S. (2007) The MARTINI force field: coarse grained model for biomolecular simulations. J. Phys. Chem. B, 111, 7812–7824. 17569554 10.1021/jp071097f

[16] Postic G., Ghouzam Y., Gelly J.-C. (2016) OREMPRO web server: orientation and assessment of atomistic and coarse-grained structures of membrane proteins. Bioinformatics, 32, 2548–2550. 27153644 10.1093/bioinformatics/btw208

[17] Hopf T.A., Colwell L.J., Sheridan R. (2012) Three-dimensional structures of membrane proteins from genomic sequencing. Cell, 149, 1607–1621. 22579045 10.1016/j.cell.2012.04.012 PMC3641781

[18] Kabsch W., Sander C. (1983) Dictionary of protein secondary structure: pattern recognition of hydrogen-bonded and geometrical features. Biopolymers, 22, 2577–2637. 6667333 10.1002/bip.360221211

[19] de Jong D.H., Singh G., Bennett W.F.D. (2013) Improved parameters for the martini coarse-grained protein force field. J. Chem. Theory Comput., 9, 687–697. 26589065 10.1021/ct300646g

[20] de Brevern A.G., Wong H., Tournamille C. (2005) A structural model of a seven-transmembrane helix receptor: the Duffy antigen/receptor for chemokine (DARC). Biochim. Biophys. Acta, 1724, 288–306. 16046070 10.1016/j.bbagen.2005.05.016

[21] Ghouzam Y., Postic G., Brevern A. G. D. (2015) Improving protein fold recognition with hybrid profiles combining sequence and structure evolution. Bioinformatics, 31, 3782–3789. 26254434 10.1093/bioinformatics/btv462

[22] Ghouzam Y., Postic G., Guerin P.-E. (2016) ORION: a web server for protein fold recognition and structure prediction using evolutionary hybrid profiles. Sci. Rep., 6, 28268. 10.1038/srep28268 PMC4913311 27319297

[23] Sippl M.J. (1990) Calculation of conformational ensembles from potentials of mena force. J. Mol. Biol., 213, 859–883. 2359125 10.1016/s0022-2836(05)80269-4

[24] Biasini M. (2014) *Zenodo*, PV-WebGL-based protein viewer.

[25] Azuara C., Lindahl E., Koehl P. (2006) PDB_Hydro: incorporating dipolar solvents with variable density in the Poisson–Boltzmann treatment of macromolecule electrostatics. Nucl. Acids Res., 34, W38–W42. 16845031 10.1093/nar/gkl072 PMC1538897

[26] Joosten R.P., Long F., Murshudov G.N. (2014) The PDB_REDO server for macromolecular structure model optimization. IUCr J., 1, 213–220. 10.1107/S2052252514009324 PMC4107921 25075342

[27] Luckey M. (2014) Membrane Structural Biology: With Biochemical and Biophysical Foundations. Cambridge University Press, Cambridge, UK.

