# Supplementary material for: TMPL: a database of experimental and theoretical transmembrane protein models positioned in the lipid bilayer
Source: Database (Oxford). 2017 Mar 24;2017:bax022. doi: 10.1093/database/bax022 (PMC5467549; doi:10.1093/database/bax022)
Supplement: Supplementary Data [file bax022_Supp.doc]

**SUPPLEMENTARY MATERIALS**


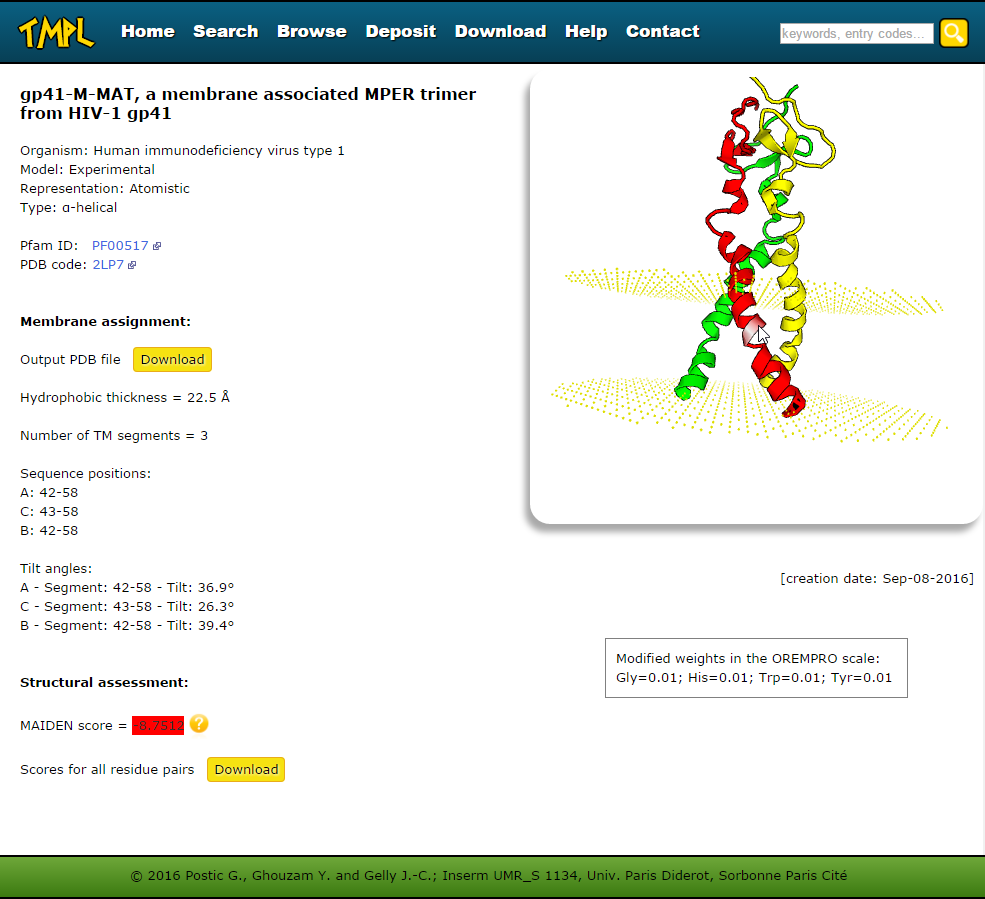


**Figure S1.** The TMPL page of the gp41-M-MAT protein structure (PDB 2lp7). For this entry, the membrane assignment has been computed using a membrane propensity scale that differs from the default binary scale: the aromatic His, Trp and Tyr, along with the achiral Gly, have been given a null weight (actually ω=0.01, because 0 is forbidden), which separates them from the other residue types (for which ω is +1.0 or −1.0), thus constituting a 'ternary' membrane propensity scale. The use of these non-default weights is indicated on the entry page. Interestingly, in the corresponding OPM assignment (<http://opm.phar.umich.edu/protein.php?pdbid=2lp7>), none of the three helices fully span the membrane, and yet the page reports three transmembrane segments.
